# A molecular assessment of ectomycorrhizal fungal communities associated with North African *Alnus glutinosa* forests

**DOI:** 10.3897/mycokeys.127.174964

**Published:** 2026-01-23

**Authors:** Oussama Saadi, Aicha Tadjine, Idriss Bouam, John Y. Kupagme, Khairiah Mubarak Alwutayd, Sten Anslan, Sergei Põlme

**Affiliations:** 1 Laboratory of Functional and Evolutionary Ecology, Faculty of Natural and Life Sciences, Chadli Bendjedid University, El Tarf, Algeria Chadli Bendjedid University El Tarf Algeria; 2 Laboratory “Biodiversity, Biotechnology, and Sustainable Development”, Faculty of Natural and Life Sciences, University of Batna 2, Fesdis 05078, Batna, Algeria University of Batna 2 Batna Algeria; 3 Mycology and Microbiology Center, University of Tartu, Tartu, Estonia University of Tartu Tartu Estonia; 4 Department of Biology, College of Science, Princess Nourah bint Abdulrahman University, Riyadh, Saudi Arabia The University of Tartu Natural History Museum and Botanical Garden Tartu Estonia; 5 Department of Biological and Environmental Science, University of Jyväskylä, Jyväskylä, Finland Princess Nourah bint Abdulrahman University Riyadh Saudi Arabia; 6 The University of Tartu Natural History Museum and Botanical Garden, Vanemuise 46, 50410, Tartu, Estonia University of Jyväskylä Jyväskylä Finland

**Keywords:** Algeria, *
Alnus
*, Diversity, Ectomycorrhizal fungi, high-throughput sequencing, Mediterranean basin

## Abstract

The diversity and biogeographic patterns of ectomycorrhizal fungi (EcMF) remain underexplored in many parts of the world, particularly in southern temperate ecosystems. Here, we present the first molecular characterization of EcMF communities associated with North African populations of *Alnus glutinosa* (L.) Gaertn., commonly known as black alder. Root samples over multiple sampling periods were collected from three sites in and around El Kala Biosphere Reserve, northeastern Algeria, and analysed using high-throughput sequencing targeting the full ITS region. We identified 101 EcMF operational taxonomic units (OTUs), representing two phyla, two classes, seven orders, 15 families, and 18 genera—predominantly Basidiomycota (98.6%). The genera *Lactarius*, *Tomentella*, and *Inocybe* consistently dominated across all sites. Community richness and diversity varied significantly among sites. Organic matter content and site identity significantly influenced EcMF community composition, whereas seasonality and other edaphic parameters showed no detectable effects. Comparative phylogenetic analysis revealed minimal overlap with EcMF communities from European, Asian, or American *Alnus* populations. These findings demonstrate that southern marginal populations of *A. glutinosa* harbour exceptionally rich and potentially unique EcMF assemblages, likely shaped by relative aridity, geographic isolation, and host lineage divergence. Our study highlights the critical importance of incorporating biogeographically peripheral ecosystems into global fungal diversity assessments, particularly in historically and environmentally distinctive regions.

## Introduction

Ectomycorrhizal fungi (EcMF) are root symbionts characterized by a Hartig net, a differentiated hyphal mantle, and an absence of intracellular colonization (Brundrett and Tedersoo 2018). These fungi are instrumental in maintaining plant health and ecosystem function, enhancing nutrient and water uptake, providing protection against pathogens and environmental stress, and mitigating heavy metal toxicity (Itoo and Reshi 2013; Policelli et al. 2020). Therefore, EcMF are increasingly being explored for their potential applications in sustainable forestry and biotechnology (Hyde et al. 2019; Niego et al. 2023).

EcMF are typically associated with woody perennials within most temperate and certain tropical tree families (Smith and Read 2008; Corrales et al. 2022). Although EcMF host plants (estimated 6,000–7,000 species) are fewer in number compared to hosts of other mycorrhizas, the diversity of EcMF themselves is far greater, encompassing an estimated 20,000–25,000 taxa (Tedersoo et al. 2010; Tedersoo and Brundrett 2017). Comprehending the diversity of EcMF has been challenging, partly due to the limitations of traditional identification methods based on morphology and anatomy (Janowski and Leski 2023). Nevertheless, the advent of molecular methods has marked a significant breakthrough in EcMF identification and diversity assessment (Horton and Bruns 2001). Despite these advancements, many understudied regions, particularly tropical and southern temperate ecosystems, remain to be explored (Tedersoo and Smith 2013).

EcMF often exhibit host specificity, ranging from generalists to specialists exclusively associating with hosts from a single lineage at various taxonomic levels, such as family, genus, or species (Vincenot and Selosse 2017). The ecological and co-evolutionary processes that determine specificity remain a topic of ongoing discussion (Molina and Trappe 1994; Bruns et al. 2002; Roy et al. 2013; Lofgren et al. 2018). The widely distributed tree genus *Alnus* Mill. (Betulaceae) is a well-known example of host-genus specificity, associating with distinct EcMF communities. For instance, fungal lineages, including monophyletic clades such as *Alpova* and *Alnicola*, exhibit strong specificity towards this tree genus (Molina 1981; Moreau et al. 2006; Tedersoo et al. 2009; Kennedy et al. 2011; Rochet et al. 2011; Moreau et al. 2013).

Among species in the *Alnus* genus, *Alnus glutinosa* (L.) Gaertn., or black alder, is native to most of Europe extending into temperate Asia and North Africa (Kajba and Gračan 2003). In North Africa, black alder populations occur at the southern limit and warmest edge of the species’ distribution range, persisting in scattered and isolated populations confined to a few coastal regions. These populations have diverged genetically from European counterparts and persist as climate relicts (Lepais et al. 2013). While *A. glutinosa* can be found in northern Libya (Kajba and Gračan 2003), northern Morocco (Valdès et al. 2002), and north-western Tunisia (Ghrabi-Gammar et al. 2009), its presence in North Africa is not as pronounced as it is within the wetland complexes of Guerbès-Senhadja and Annaba-El Kala, located in north-eastern Algeria (Belouahem-Abed et al. 2011). In these wetlands, *Alnus glutinosa* grows on hydromorphic soils with variable organic matter and moisture regimes, ranging from alluvial sands to peaty substrates, which experience pronounced seasonal fluctuations, and occupies a mosaic of fluvial, swampy, and riparian forest habitats increasingly altered by human activities (Belouahem-Abed et al. 2011; Necer et al. 2019).

Extensive research has explored the EcMF communities associated with black alder in European and Asian populations, consistently indicating a relatively species-poor assemblage dominated by genera such as *Tomentella*, *Alnicola*, *Lactarius*, and *Alpova* (Tedersoo et al. 2009; Rochet et al. 2011; Põlme et al. 2013; Roy et al. 2013; Thiem et al. 2018). However, a conspicuous knowledge gap exists regarding the EcMF communities of North African black alder populations, which remain virtually unexplored (Põlme et al. 2013). Addressing this research gap is imperative for understanding the global diversity and distribution of *Alnus*-associated EcMF, especially considering that various local environmental factors, such as soil parameters, habitat conditions, seasonal variations, geographic isolation, and genetic divergence within the host’s distribution range, have been proposed as key determinants of the structure and diversity of these communities (Becerra et al. 2005b; Tedersoo et al. 2009; Kennedy and Hill 2010; Roy et al. 2013).

Here, we investigated the EcMF communities associated with *Alnus glutinosa* in three locations in northeastern Algeria. We carried out a concurrent assessment of EcMF across all four seasons and quantified soil properties at each site to assess potential correlations with the structure of EcMF community. Given that these Algerian populations occupy the warmest and geographically isolated edge of the species’ range and have diverged genetically from other populations, we hypothesize that their EcMF assemblages may exhibit distinctive patterns of structure and diversity. This study therefore aims to: (1) document the EcMF community diversity and structure associated with North African black alder populations; (2) compare the observed diversity and composition with previously published data from Europe and Asia to establish the biogeographical distinctiveness of the North African assemblage; and (3) identify the soil properties influencing the local community structure.

## Methods

### Study area

This study was conducted in El Kala Biosphere Reserve (hereafter KBR) and its surrounding areas, located in El Tarf Province, northeastern Algeria (Suppl. material 1: fig. S1). KBR covers an area of 76,384 ha and is situated within the East Algeria biodiversity hotspot in the Mediterranean Basin (Myers et al. 2000). In the wetland complexes of KBR, black alder trees predominantly compose the tree canopy of the existing swamp forests (Kahli et al. 2018). Within this region, we identified three accessible black alder forest sites: El Mellah, Righia, and Verges. These sites feature pure, old-growth stands of *A. glutinosa*, also referred to as *Alnetum* communities (Claessens et al. 2010). The distances between these sampling sites range from 7 to 16 km. We obtained elevational and climatic data for these sites from WorldClim (version 2.1), which offers a spatial resolution of 30 arc-seconds (Fick and Hijmans 2017). All three sampling sites are situated at low elevations (less than 20 m above sea level) and are characterized by a humid Mediterranean climate with temperate to warm winters (as based on Emberger quotient; Daget 1977), with an annual mean precipitation ranging from 875 to 904 mm.

### Sampling protocol

At each site, five *Alnus glutinosa* trees were randomly selected during each survey, ensuring they were at least 10 m apart to minimize spatial correlation among the samples (Lilleskov et al. 2004). Trees selected in one survey were not necessarily the same as those sampled in other surveys. Around each tree, we collected one soil core (5 cm diameter × 25 cm depth) within a one-meter radius of the trunk. Root samples were extracted from the same 25 cm depth. Following collection, the roots were placed in plastic bags and stored at -4 °C for subsequent processing. Sampling was conducted once per season, specifically in December, March, June, and September, yielding a total of 60 root samples (5 trees × 3 sites × 4 seasons). Soil samples were homogenized and pooled by site and season for edaphic physico-chemical analyses, resulting in a total of 12 samples (3 sites × 4 seasons).

### Soil analyses

Soil samples were air-dried, sieved through a 2-mm sieve, and the fraction finer than or equal to 2 mm was analysed. Electrical conductivity (EC) and pH were measured for each soil sample in a 1:5 soil/water mixture using a conductance meter and pH meter, respectively. Organic matter (OM) content was determined using the Nelson and Sommers (1996) method. Total lime (TL) was determined using volumetric calcimetry. Practical salinity (PS) was derived from EC following Fofonoff and Millard (1983). These edaphic variables have previously been identified as major drivers of EcMF composition, particularly in *Alnus*-associated habitats (e.g., Becerra et al. 2005b; Tedersoo et al. 2014; Sun et al. 2016; Kjøller et al. 2017; Thiem et al. 2018).

### Molecular analyses for root samples

Prior to homogenization, root samples were dried at 35 °C for 24 h. DNA from *Alnus* roots was extracted from 0.25 g of roots from each composite sample using the PowerMax Soil DNA Isolation kit (Qiagen). Before extraction, roots were pulverized by bead beating using Retsch MM400 mixer mill (Retsch, Haan, Germany), weighed, and 520 μl of proteinase K (600 units per ml) was added to the samples. The full ITS region (ITS1-5.8S-ITS2) was amplified via PCR using the primer pair ITS9mun and ITS4ngsuni (Tedersoo and Anslan 2019). PCR reactions contained the following components: 5 µl of 5× HOT FIREPol Blend Master Mix, 0.5 µl of each 20 mM primer, 1 µl of DNA extract, and 18 µl of ddH_2_O. The cycling conditions were as follows: initial denaturation at 95 °C for 15 min, followed by 30 cycles of 95 °C for 30 s, 55 °C for 30 s, and 72 °C for 1 min, with a final extension at 72 °C for 10 min, and a hold at 4 °C. PCR products from duplicate reactions were pooled and visualized on a 1% agarose gel to ensure appropriate amplicon size (600–800 bp). Negative controls were implemented to detect potential contamination, which showed no detection of contaminants based on the gel electrophoresis image. Pooled amplicons were purified with FavorPrepTM kit (Favorgen) and sent to the Norwegian Sequencing Centre for PacBio SMRTbell library preparation and sequencing on a Sequel II instrument.

### Bioinformatics

Raw PacBio sequences were transformed to an operational taxonomic unit (OTU) table using PipeCraft2 (v0.1.4; Anslan et al. 2017) metabarcoding data processing software, which wraps the following tools at each step. Cutadapt v3.5 (Martin 2011) was used for demultiplexing (allowed 1 mismatch and overlap of 11 bp to tag sequence) and clipping primers (allowed 2 mismatches, and overlap of 18 bp). Quality filtering was performed by allowing maximum error rate of 1 per sequence and no ambiguous bases using VSEARCH (v2.18.0; Rognes et al. 2016) as implemented in PipeCraft2. Putative chimeric sequences were removed with “uchime_denovo” method in VSEARCH. The sequences were subsequently passed through ITSx v1.1.3 (Bengtsson-Palme et al. 2013) to extract full ITS region without conservative gene fragments (with default settings, except E = 1^e-2^). ITS sequences were clustered into OTUs using VSEARCH (--iddeff = 2, similarity threshold of 98%), and post-clustered using LULU (v.0.1.0; Frøslev et al. 2017) merge potential “daughter-OTUs” (with default settings, except minimum match = 98, minimum relative cooccurrence = 0.8). The match list for LULU was generated using BLASTn (Camacho et al. 2009). Taxonomy was assigned to filtered OTUs using BLASTn search against UNITE v9 database (Nilsson et al. 2019; Abarenkov et al. 2022). Only matches with a minimum sequence identity of 90% and alignment coverage greater than 20% were retained for taxonomic assignments.

The raw sequencing data are deposited in European Nucleotide Archive (ENA) at EMBL-EBI under accession number PRJEB94942 (https://www.ebi.ac.uk/ena/browser/view/PRJEB94942).

### Statistical analyses

All statistical analyses were performed in the RStudio interface (version 2023.3.0.386) (Posit team 2023) of the R software (version 4.2.3) (R Core Team 2023). To compare our ectomycorrhizal fungal sequences with other studies focusing on *Alnus* associated fungi (i.e., Kennedy et al. 2011; Põlme et al. 2013), we conducted a phylogenetic clustering analysis using the ‘ape’ package (Paradis and Schliep 2019). Published sequences were aligned with our entire dataset of ectomycorrhizal sequences, and a genetic distance matrix was computed (Kimura 2-parameter model). Sequences were clustered into OTUs at 98% similarity (hclust, complete linkage), following standard fungal species delineation (e.g., UNITE’s ITS criterion).

To assess the effectiveness of the sampling protocol and the adequacy of sampling effort and sequencing depth, we evaluated the completeness of the sampling through sample coverage for each site using the site’s overall dataset, with each tree treated as a separate sampling unit (Chao and Jost 2012). For all R/E curves, 500 replicate bootstrapping runs were performed to estimate 95% confidence intervals, using the “iNEXT” package (Hsieh et al. 2016).

Differences in OTUs richness and diversity of EcMF communities across the three sites were assessed using Linear Mixed Models (LMMs) in the “lme4” (Bates et al. 2015) package. For the richness analyses, we computed the standardized residuals of the number of OTU counts relative to the logarithm of the number of sequences obtained. This was done to account for differences in sequencing depth (Tedersoo et al. 2014). In assessing diversity, we opted for the Shannon diversity index (Haegeman et al. 2013). Tree-level OTU richness residuals and Shannon diversity values were treated as response variables, with site as a fixed effect and season as a random intercept to account for potential temporal variation among sampling periods. Significance was determined using a Type III Analysis of Variance (ANOVA). If the results were significant, we conducted post-hoc pairwise comparisons using Tukey’s all-pair comparisons. To ensure the assumptions of the models were met, we performed residual diagnostics with the “DHARMa” package (Hartig 2022). The validation of the models and the results are presented in Suppl. material 1: fig. S2.

Differences in all measured soil physico-chemical properties, each treated as a response variable, among sites, considering all samples within each site, and among seasons, considering all samples within each season, were assessed using the Kruskal-Wallis test, a non-parametric alternative to ANOVA applied because the assumptions of normality and homogeneity of variance were not met. Where significant differences were identified, post-hoc pairwise comparisons were performed using Dunn’s test with Bonferroni correction for multiple testing (Dinno 2015).

Non-metric multidimensional scaling (NMDS) was conducted to visually investigate the variation in EcMF community structure across site × season combinations. All measured soil properties were fitted to the NMDS ordination using 999 permutations with the “envfit” function. The effect of site, season, and soil properties on the EcMF community was subsequently analysed using permutational multivariate analysis of variance (PERMANOVA; Anderson 2014) with 999 permutations using the “adonis2” function. As PERMANOVA can be sensitive to heterogeneity in dispersions, we employed the “betadisper” function to examine the homogeneity of dispersion across groups. Both NMDS and PERMANOVA were based on Hellinger-transformed sequence abundance data for each OTU and the Bray-Curtis dissimilarity index, and were performed using the “vegan” package (Oksanen et al. 2022).

## Results

**Figure 1.**
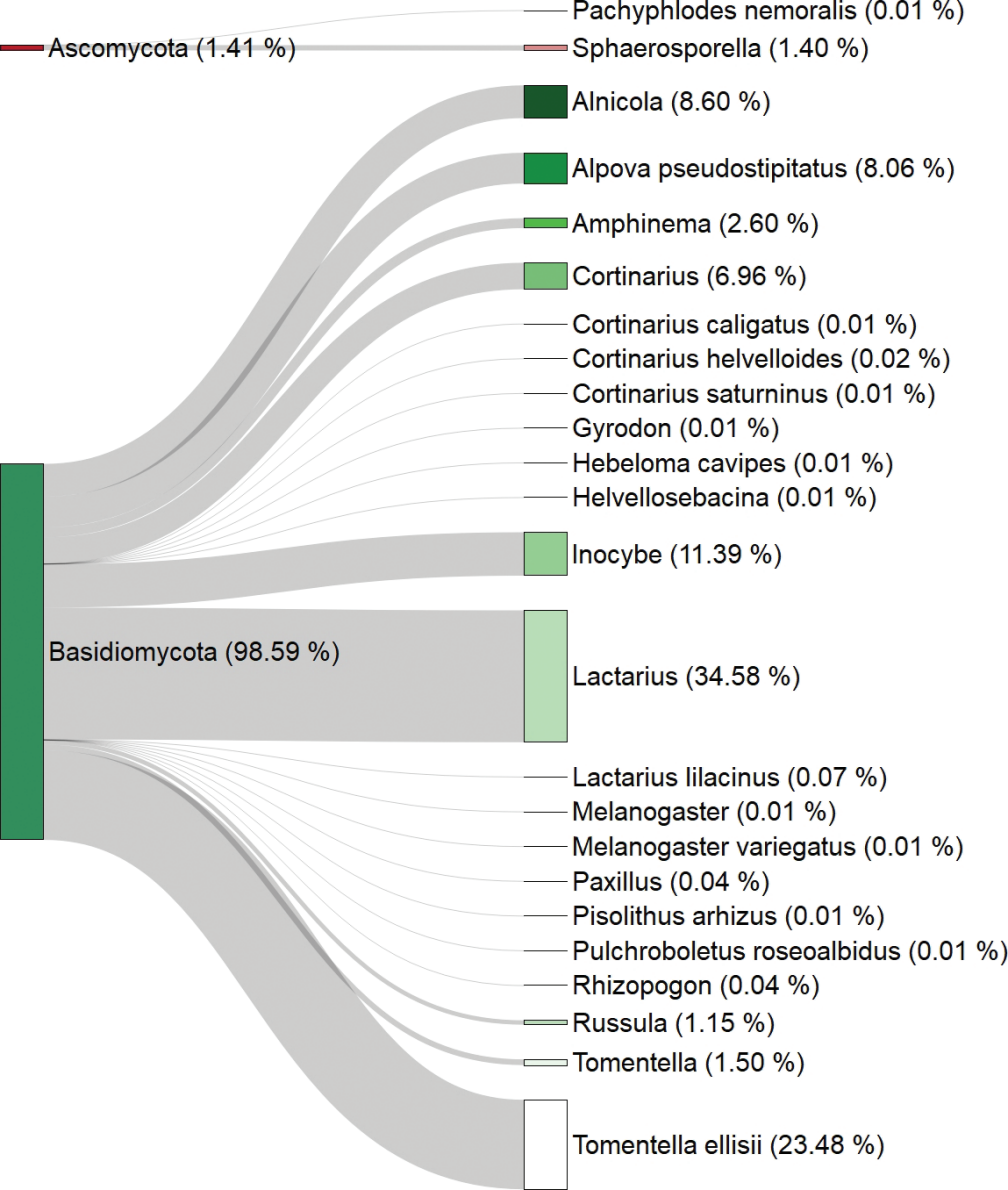
Sankey diagram illustrating the relative abundance of ectomycorrhizal fungi (EcMF) operational taxonomic units (OTUs), denoted in parentheses, across taxonomic levels. Abundances are based on the total number of sequencing reads pooled across all 60 root samples collected from the three study sites. Flow widths are proportional to sequence abundance. Taxonomic assignments are displayed at the genus or species level, whenever possible.

Overall, the EcMF community associated with *Alnus glutinosa* roots in KBR was composed of 101 OTUs belonging to two phyla, two classes, seven orders, 15 families, and 18 genera (Suppl. material 2: table SS1). The majority of these OTUs (99) belonged to the phylum Basidiomycota, which also accounted for 98.59% of the sequences, while the remaining OTUs belonged to the phylum Ascomycota. The most abundant OTUs (>10% of sequences) belonged to the genera *Lactarius*, *Tomentella*, and *Inocybe*, which together accounted for over 71% of the sequence abundance (Fig. 1). Sequencing depth averaged 13,682 reads per sample after demultiplexing. Sample completeness values were 0.87 for El Mellah, 0.79 for Righia, and 0.89 for Verges, reflecting satisfactory sample coverage and adequate sequencing depth (Suppl. material 1: fig. S3).

**Figure 2.**
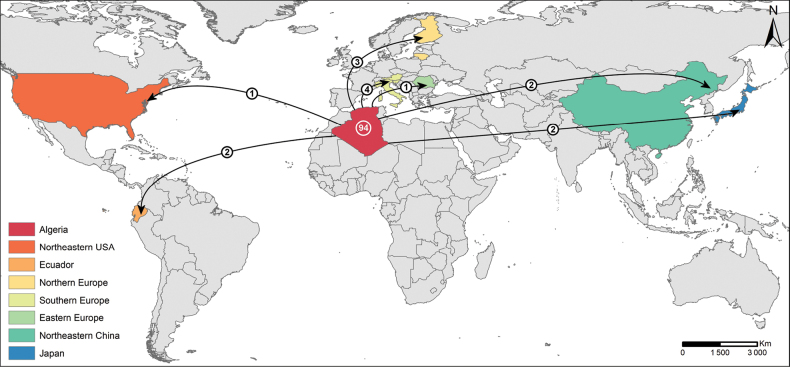
Map showing the number of unique ectomycorrhizal fungi (EcMF) operational taxonomic units (OTUs) associated with *Alnus glutinosa* in Algeria (white number) and the number of shared OTUs between Algeria and other regions/countries (indicated by arrows, with OTU counts labelled). The comparison is based on the complete dataset of 101 EcMFOTUs recovered from the three study sites and previously published *Alnus*-associated datasets (i.e., Kennedy et al. 2011; Põlme et al. 2013). Shared OTUs are shown for Northern Europe (Finland, Lithuania), Southern Europe (Austria, Italy, Slovenia), Eastern Europe (Romania), Northeast USA, Ecuador, Northeast China, and Japan.

Phylogenetic comparison with global datasets revealed 94 OTUs exclusive to Algeria. OTU sharing with other regions was minimal and included four genera, namely *Cortinarius*, *Inocybe*, *Lactarius*, and *Tomentella*. Notably, one OTU was shared with the Northeast USA, two with Ecuador, three with Northern Europe (Finland, Lithuania), four with Southern Europe (Austria, Italy, Slovenia), one with Eastern Europe (Romania), and two each with Northeast China and Japan (Fig. 2). In terms of the number of sequences, shared OTUs represented 67.5%, while unique OTUs hosted 32.5% of the sequences in the current study. Restricting the analysis to the dominant ectomycorrhizal genera in terms of sequence abundance, namely *Lactarius*, *Tomentella*, and *Inocybe*, increased the proportion of shared OTUs to 83.6%, with 16.4% remaining unique.

**Figure 3.**
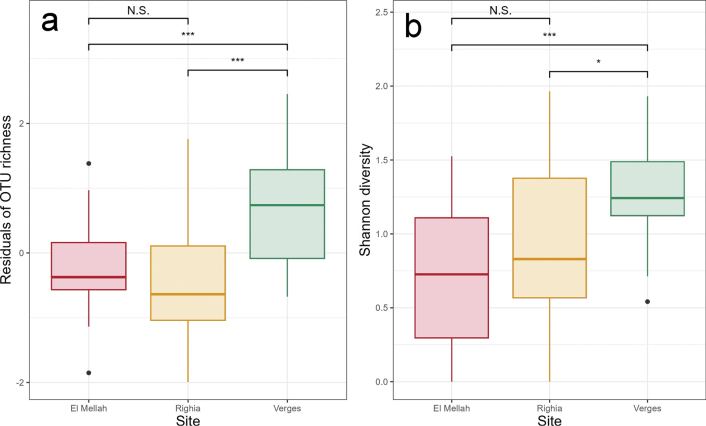
(**a**) Residual operational taxonomic unit (OTU) richness (as a measure of alpha diversity), and (**b**) Shannon diversity of ectomycorrhizal fungi (EcMF) communities across the three study sites (N = 20 tree root samples per site). Black dots indicate outliers. Statistically significant differences among sites were assessed using Linear Mixed Models (LMM) with season as a random effect, followed by Tukey’s post-hoc comparisons. Significance levels are indicated as follows: N.S. not significant, ** *p* < 0.01, *** *p* < 0.001.

Verges harbored the highest number of unique EcMFOTUs (23), followed by Righia (22) and El Mellah (8). Fifteen OTUs were shared among all three sites, while pairwise overlaps included 14 OTUs between El Mellah and Verges, 10 between Righia and Verges, and 9 between El Mellah and Righia (Suppl. material 1: fig. S4). The results from the LMMs demonstrated that Verges had the highest mean OTU richness and diversity. Significant differences in both richness and diversity were observed between Verges and the other two sites, El Mellah and Righia (Fig. 3).

Soil properties across all three sites and the results of the Kruskal-Wallis test are summarized in Suppl. material 3: table S2. Most edaphic variables exhibited minimal spatial or temporal variation; Kruskal-Wallis tests showed no significant differences among sites or between seasons for pH, electrical conductivity, total lime, or practical salinity. A notable variation was found in the content of organic matter, with Righia exhibiting significantly higher values than both El Mellah and Verges. However, the Dunn’s test revealed significant differences only between Righia and Verges (Suppl. material 3: table S3). There were no significant differences observed in any soil properties between seasons.

**Figure 4.**
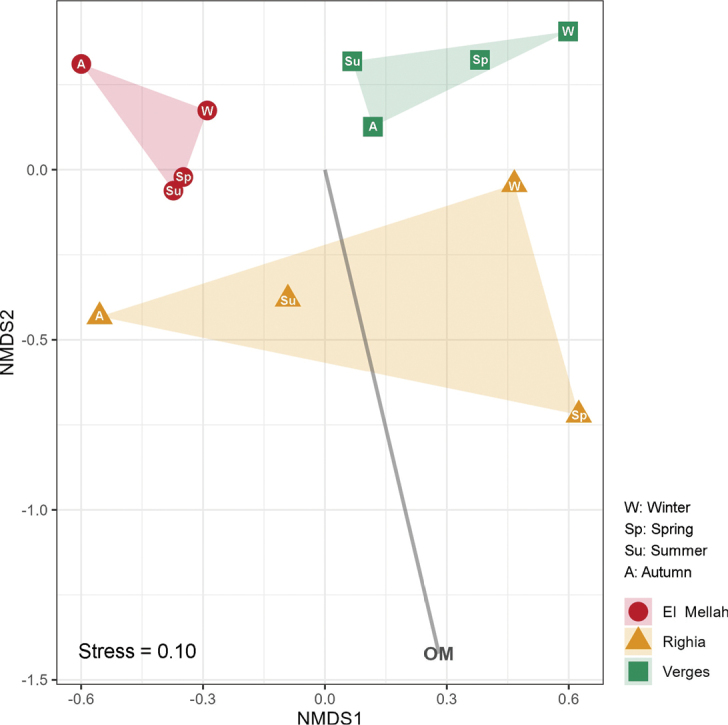
Non-metric multidimensional scaling (NMDS) ordination of the ectomycorrhizal fungi (EcMF) community structure across the three study sites (N = 20 tree root samples per site) based on Bray-Curtis dissimilarity of Hellinger-transformed operational taxonomic units (OTU) abundance data. Shapes represent centroids of EcMF community structure for each site × season combination and convex hulls encompass the four seasonal centroids within each site. The soil variable organic matter (OM), which showed a significant correlation with the ordination is displayed, with the length and orientation of the arrow indicating the strength and direction of the correlation.

The NMDS ordination plot (Fig. 4) yielded a good stress score of 0.10, revealing a distinct separation among the three sites, thus indicating site-specific differences in the EcMF community structure. A significant correlation was also observed between the organic matter content and the ordination (Fig. 4; Suppl. material 3: table S4). The PERMANOVA results (Table 1) corroborated these findings, demonstrating that both the site and organic matter content significantly influenced the EcMF community structure, although the effect of organic matter was less pronounced. The site accounted for 43.3% of the observed variation, while the organic matter content explained 19.0%. Other factors tested, namely season, electrical conductivity, pH, total lime, and practical salinity, did not significantly influence the EcMF community structure. The assessment of homogeneity of dispersion across groups yielded no significant results.

**Table 1.** PERMANOVA and Betadisper results assessing the effects of site, season, and soil properties on ectomycorrhizal fungi (EcMF) community structure (N = 20 tree-level root samples per site, based on Bray-Curtis dissimilarity of Hellinger-transformed operational taxonomic units (OTU) abundances). Significant differences are indicated in bold. n.a. indicates not available.

	**PERMANOVA**				**Betadisper**		
	**df**	***Pseudo-F***	***R^2^***	***P***	**df**	***F***	***P***
Site	2	3.437	0.433	<0.001***	2	2.456	0.140
Season	3	0.767	0.223	0.760	3	1.718	0.240
Electrical conductivity	1	0.434	0.041	0.933	11	n.a.	n.a.
pH	1	0.871	0.080	0.541	11	n.a.	n.a.
Total lime	1	1.209	0.107	0.292	11	n.a.	n.a.
Organic matter	1	2.355	0.190	0.022*	11	n.a.	n.a.
Practical salinity	1	0.432	0.041	0.931	11	n.a.	n.a.

## Discussion

This study provides the first characterization of EcMF communities associated with *Alnus glutinosa* populations in North Africa, substantially expanding our understanding of the biogeography of *Alnus*-associated EcMF. While the structure and composition of these communities have been previously studied in European and Asian populations of *A. glutinosa* (Tedersoo et al. 2009; Põlme et al. 2013; Roy et al. 2013; Thiem et al. 2018), our work addresses an unexplored region, contributing valuable data to the broader biogeographic framework of these fungal communities.

We identified 101 OTUs of EcMF associated with *A. glutinosa* root tips in Algeria, representing one of the highest regional diversities reported for *Alnus*-associated EcMF communities. This richness markedly exceeded values from comparable studies: five times greater than *A. glutinosa* in Estonia (18 OTUs; Tedersoo et al. 2009), four times greater than *A. rubra* in the USA (22 OTUs; McBurney et al. 2017), and 15% higher than the cumulative diversity across five *Alnus* species in France (86 OTUs; Roy et al. 2013). It was, however, lower than the 146 OTUs reported globally across 22 *Alnus* species (Põlme et al. 2013), the 175 OTUs recovered from *A. rubra* in USA (Kennedy et al. 2014), and the 275 OTUs documented from *A. glutinosa* in Belgium (Boeraeve et al. 2019). These differences in recorded diversity could, in part, reflect methodological differences across studies, including the use of Sanger sequencing from single root tips in earlier works, variations in the bioinformatic processing of the high-throughput sequencing data, as well as variations in sampling methods (Janowski and Leski 2023).

Sample-size-based rarefaction showed that our sampling captured 79–89% of estimated OTU diversity, with only nine singleton OTUs. Notably, these singletons included genera classically associated with *Alnus* (*Alnicola*, *Cortinarius*, *Inocybe*, *Lactarius*, and *Tomentella*) (Rochet et al. 2011), suggesting minimal undersampling of dominant taxa. Together, these results indicate that the observed diversity robustly reflects the actual EcMF richness in Algerian *A. glutinosa* forests.

Consistent with global patterns in *Alnus*-associated EcMF communities (e.g., Rochet et al. 2011; Thiem et al. 2018), Basidiomycota represented the dominant phylum in our study. The three most abundant genera, namely *Lactarius*, *Tomentella*, and *Inocybe*, mirrored the dominance patterns reported by Rochet et al. (2011), with *Lactarius* and *Tomentella* consistently ranking among the most frequent taxa across *Alnus* forests. Notably, while *Inocybe* was relatively highly abundant in our studied sites, it has been documented only occasionally in other *Alnus*-associated communities (Rochet et al. 2011). However, distinct dominance patterns have been reported elsewhere; for instance, Kennedy et al. (2011) observed an unusually high abundance of *Clavulina* in *Alnus* stands in Mexico, and high frequencies of Ascomycetes were reported in Iranian *Alnus* forests (Põlme et al. 2013). This variability suggests that while a limited number of EcMF genera tend to dominate *Alnus* ecosystems globally (Roy et al. 2013), regional shifts in dominant taxa also occur. The ecological drivers of such patterns remain unclear, though founder effects may play a role (Douhan et al. 2011). Additionally, we note that three EcMF genera detected in our study, namely *Amphinema*, *Melanogaster*, and *Rhizopogon*, are typically associated with *Quercus* spp. and *Pinus* spp. hosts (Sulzbacher et al. 2016; Mandolini et al. 2024; Yuan et al. 2024). Within KBR, *Quercus canariensis* is characteristic of riparian forests, whereas *Q. suber* and *Pinus pinaster* dominate dry forests (Kahli et al. 2018). The detection of these genera in our dataset may therefore reflect spore deposition or propagule input from nearby oak and pine stands rather than active ectomycorrhizal symbioses with *Alnus glutinosa*, consistent with the low-abundance occurrences often observed outside primary host stands (Taylor and Bruns 1999; Nara 2009; Peay et al. 2012).

A striking 93% of the 101 OTUs (94 OTUs) detected in our study were unique to Algeria, with shared OTUs limited to four genera, namely *Cortinarius*, *Inocybe*, *Lactarius*, and *Tomentella*, and minimal overlap (1–4 OTUs) with other regions or countries. It should be noted, however, that “unique OTUs” here refers to uniqueness with respect to these datasets and does not necessarily imply biological endemism, which remains to be determined through broader sampling and phylogenetic analyses (Tedersoo et al. 2022). Although most OTUs were unique in this sense, the community composition, in terms of relative sequence abundance, was dominated by a subset of globally shared taxa, particularly *Lactarius*, *Tomentella*, and *Inocybe*. Assuming that the higher relative sequence abundance may reflect roughly higher dominance (Lamb et al. 2019), this suggests that while Algerian *Alnus*-associated EcMF communities are highly distinctive, their overall composition is still largely dominated by widely distributed taxa. Such a pattern reflects the regionally conserved yet partially shared structure of *Alnus*-associated EcMF communities previously reported by Roy et al. (2013). Several non-exclusive factors may underlie this distinctiveness. In their investigation of *Alnus*-associated EcMF, Tedersoo et al. (2009) observed that drier plots supported a greater number of unique taxa than wetter sites. More recently, Tedersoo et al. (2022) demonstrated that fungal endemism, including that of *Alnus*-associated EcMF, is globally widespread, with few taxa exhibiting cosmopolitan distributions. In agreement with these global patterns, our study, conducted at the southernmost and climatically driest margin of *A. glutinosa*’s global distribution (Kajba and Gračan 2003), revealed an exceptionally species-rich assemblage, characterized by a high proportion of potentially unique taxa. This suggests that increased aridity at the ecological margins of *A. glutinosa* may promote both EcMF diversity and uniqueness. Additionally, geographic isolation likely plays an important role. North African *A. glutinosa* populations represent genetically distinct relicts from the Last Glacial Maximum, exhibiting marked divergence from European populations (Lepais et al. 2013). Given that phylogenetic relationships among host plants and geographical connectivity have been shown to influence *Alnus*-associated EcMF community composition (Põlme et al. 2013), such historical and biogeographic factors could contribute to the distinct fungal assemblages observed. Furthermore, local environmental factors, including soil properties, habitat structure, and seasonal dynamics, are recognized drivers of EcMF distribution patterns (Becerra et al. 2005b; Kennedy and Hill 2010; Põlme et al. 2013) and may also be pivotal in shaping the unique EcMF of North African *A. glutinosa* stands. While these mechanisms offer plausible explanations, future studies integrating comprehensive environmental metadata, host genetic data, and expanded sampling across North Africa will be essential to disentangle their relative contributions to the assembly and biogeography of *Alnus*-associated ectomycorrhizal communities.

Significant differences in OTU richness and diversity were detected among the three study sites, with particularly elevated mean values at Verges compared to the other two locations. Despite this variability, all sites were consistently dominated by fungal genera commonly associated with *Alnus* species worldwide, notably *Alnicola*, *Alpova*, *Cortinarius*, *Lactarius*, *Inocybe*, and *Tomentella* (Rochet et al. 2011). Among the measured soil variables, only organic matter content had a significant effect on EcMF community structure, whereas soil pH, electrical conductivity, total lime, and practical salinity showed no discernible influence. Such a pattern is not unexpected, as these parameters exhibited minimal spatial variation across the study sites. Additionally, no significant seasonal effect on community composition was detected, suggesting temporal stability in the structure of *Alnus*-associated EcMF assemblages over the sampling period. Nonetheless, a pronounced site effect was also evident, and given the limited number of sites, environmental effects cannot be fully disentangled from site-specific influences. Even so, the observed association with organic matter is congruent with patterns previously reported in *Alnus*–EcMF systems across other regions, reinforcing its potential role as an important ecological driver. For example, Becerra et al. (2005a, b) reported similar patterns in *A. acuminata* in Argentina, and Tedersoo et al. (2009) found organic matter to influence EcMF colonization in *A. glutinosa* and *A. incana* in Estonia. Importantly, Tedersoo et al. (2009) also emphasized, consistent with our findings, that the site exerts the strongest influence on EcMF community structure. Despite the site-specific effect, the observed combination of high organic matter at Righia and its lower OTU richness aligns with findings in other *Alnus* systems, where EcMF colonization and richness can decrease in sites with high organic matter (Harvey et al. 1981). This suppression of richness by organic matter may reflect selective environmental filtering, as the accumulated organic matter in *Alnus* stands is typically high in nitrogen, favouring dominant EcMF taxa (Becerra et al. 2005a). Recent research further indicates that the relationship between EcMF communities and soil organic matter is complex and context-dependent, involving multiple ecological processes and feedbacks (Lindahl and Tunlid 2015; Wang et al. 2025).

Our findings reveal that North African *Alnus glutinosa* forests harbor an exceptionally diverse and potentially unique EcMF community. Importantly, this study underscores the importance of investigating EcMF communities in underexplored regions, where environmental and historical factors may foster unique fungal assemblages. Further research integrating expanded geographic sampling and environmental data across North Africa will be essential to disentangle the ecological and biogeographic processes shaping these communities.

## Additional information

### Use of AI

No use of AI was reported.

### Author ORCIDs

Oussama Saadi https://orcid.org/0009-0006-8785-9230

Idriss Bouam https://orcid.org/0000-0002-4394-9297

John Y. Kupagme https://orcid.org/0000-0002-9981-050X

Khairiah Mubarak Alwutayd https://orcid.org/0000-0001-7071-8710

Sten Anslan https://orcid.org/0000-0002-2299-454X

Sergei Põlme https://orcid.org/0000-0002-9658-1166

### Data availability

All of the data that support the findings of this study are available in the main text or Supplementary Information.

## Supplementary materials

Supplementary material 1 Supplementary figures Data type pdf Explanation note **fig. S1**. Geographic location of the study sites: El Mellah (M), Righia (R), and Verges (V) within El Tarf Province, northeastern Algeria. **fig. S2**. Results of the residual diagnostic tests of the Linear Mixed Models (LMMs) testing for the effect of site on (A) residuals of operational taxonomic units (OTUs) richness, and (B) Shannon diversity index. **fig. S3**. Sample-size-based rarefaction and extrapolation curves of the sample coverage across the three sites. **fig. S4**. Venn diagram illustrating the distribution of ectomycorrhizal fungi (EcMF) operational taxonomic units (OTUs) among the three study sites: El Mellah, Righia, and Verges. https://binary.pensoft.net/file/1518264This dataset is made available under the Open Database License (http://opendatacommons.org/licenses/odbl/1.0/). The Open Database License (ODbL) is a license agreement intended to allow users to freely share, modify, and use this Dataset while maintaining this same freedom for others, provided that the original source and author(s) are credited. Oussama Saadi, Aicha Tadjine, Idriss Bouam, John Y. Kupagme, Khairiah Mubarak Alwutayd, Sten Anslan, Sergei Põlme

Supplementary material 2 Sampling metadata, edaphic variables, and ectomycorrhizal fungi (EcMF) operational taxonomic units (OTUs) taxonomy for North African Alnus glutinosa forests Data type xlsx https://binary.pensoft.net/file/1518265This dataset is made available under the Open Database License (http://opendatacommons.org/licenses/odbl/1.0/). The Open Database License (ODbL) is a license agreement intended to allow users to freely share, modify, and use this Dataset while maintaining this same freedom for others, provided that the original source and author(s) are credited. Oussama Saadi, Aicha Tadjine, Idriss Bouam, John Y. Kupagme, Khairiah Mubarak Alwutayd, Sten Anslan, Sergei Põlme

Supplementary material 3 Supplementary tables Data type docx Explanation note **table S2**. Mean values ± standard deviation and range of measured soil properties across the three study sites (N = 3 pooled soil samples per site per season). **table S3**. Results of Dunn’s test for pairwise comparisons of pH and organic matter between the three study sites. **table S4**. Correlation coefficients and significance values for soil variables and non-metric multidimensional scaling (NMDS) axes. https://binary.pensoft.net/file/1518266This dataset is made available under the Open Database License (http://opendatacommons.org/licenses/odbl/1.0/). The Open Database License (ODbL) is a license agreement intended to allow users to freely share, modify, and use this Dataset while maintaining this same freedom for others, provided that the original source and author(s) are credited. Oussama Saadi, Aicha Tadjine, Idriss Bouam, John Y. Kupagme, Khairiah Mubarak Alwutayd, Sten Anslan, Sergei Põlme
