# Supplementary figures and images for: A molecular assessment of ectomycorrhizal fungal communities associated with North African Alnus glutinosa forests
Source: MycoKeys. 2026 Jan 23;127:169–89. doi: 10.3897/mycokeys.127.174964 (PMC12859642; doi:10.3897/mycokeys.127.174964)

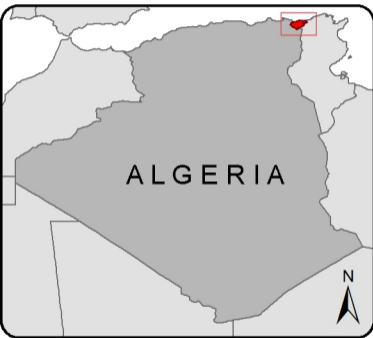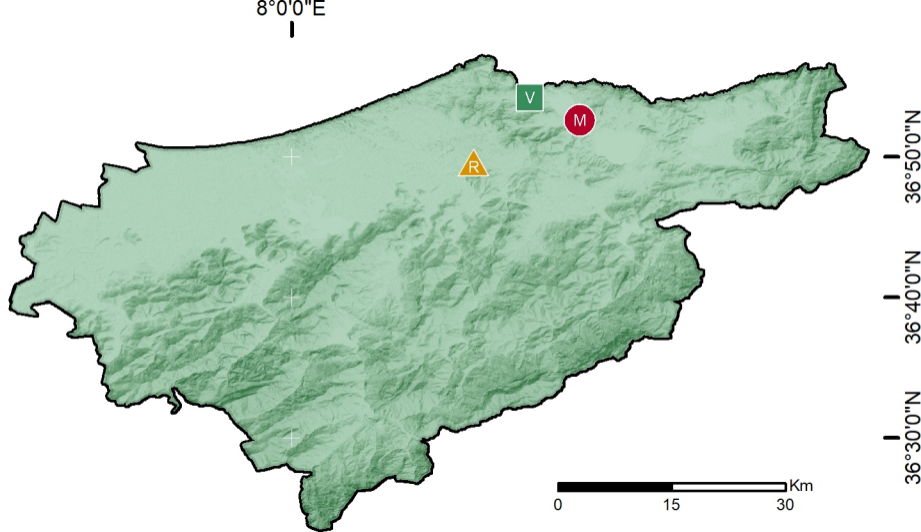

**a**

DHARMA residual

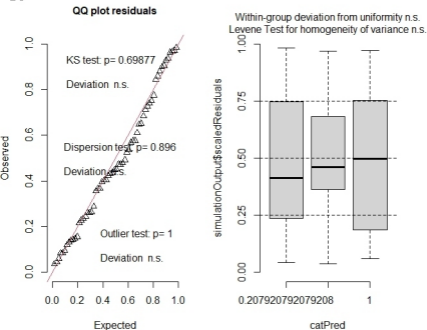**b**

DHARMA residual

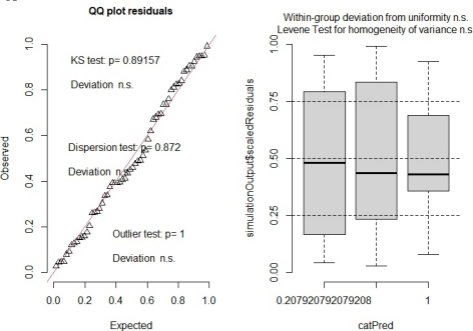

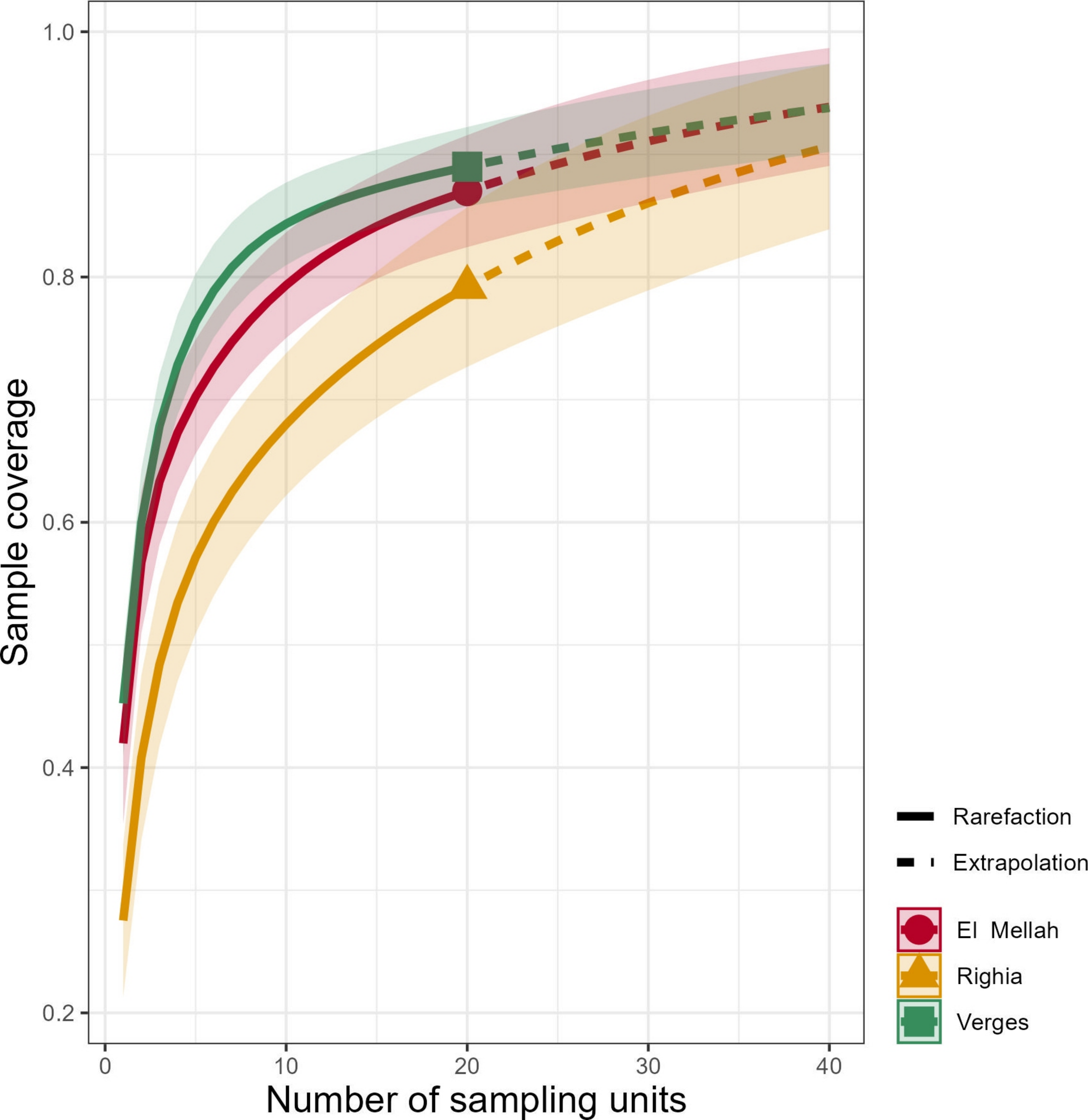

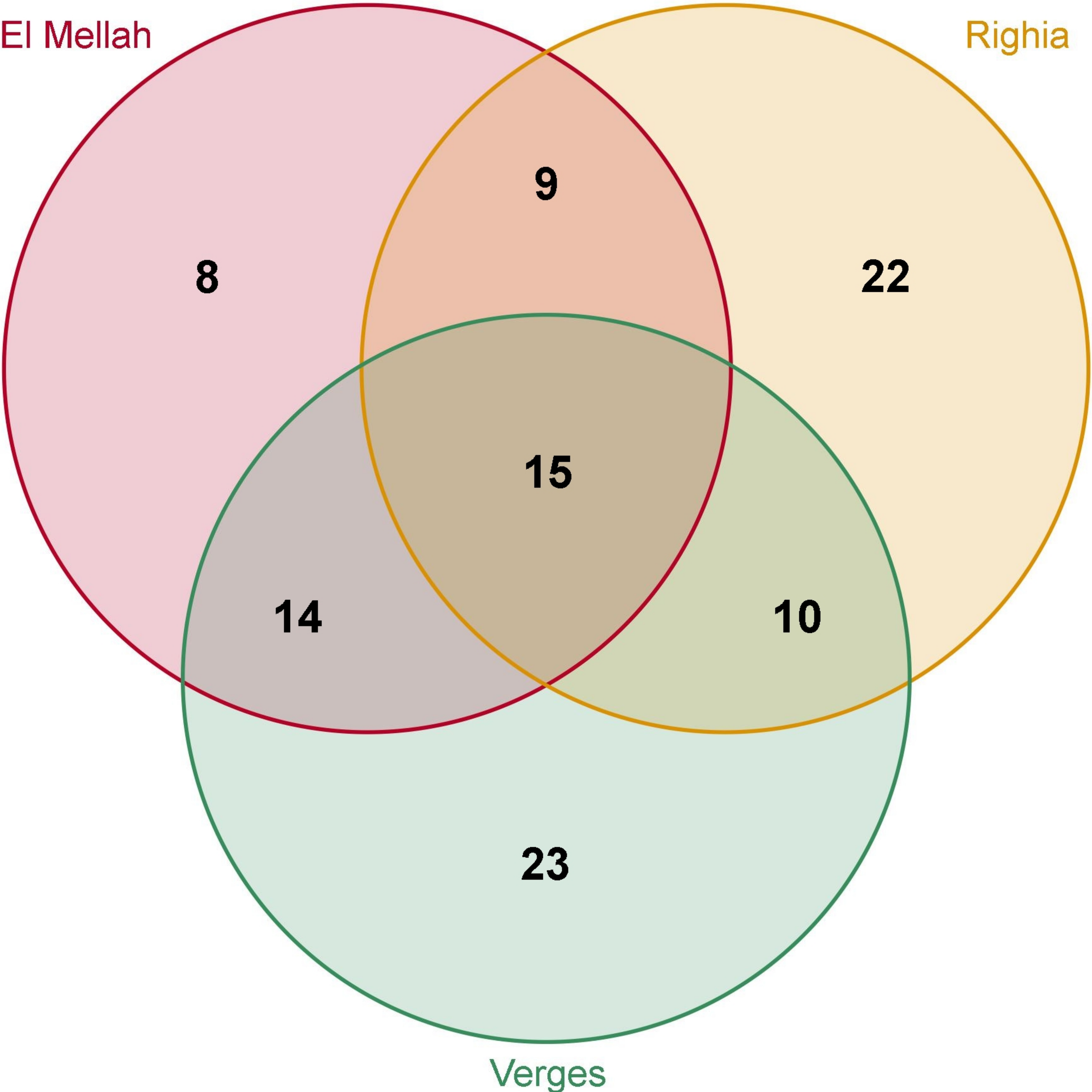

Supplement: Supplementary material 1 — Supplementary figures [file mycokeys-127-169-s001.pdf]
