# Supplementary material for: A molecular assessment of ectomycorrhizal fungal communities associated with North African Alnus glutinosa forests
Source: MycoKeys. 2026 Jan 23;127:169–89. doi: 10.3897/mycokeys.127.174964 (PMC12859642; doi:10.3897/mycokeys.127.174964)
Supplement: Supplementary material 3 — Supplementary tables [file mycokeys-127-169-s003.docx]

**Table S2.** Mean values ± standard deviation and range of measured soil properties across the three study sites (N = 3 pooled soil samples per site per season). The results of Kruskal-Wallis tests indicate the effects of site and season on each soil property. Significant differences are indicated in bold.

|  | **Unit** | **El Mellah** | **Righia** | **Verges** | **Kruskal-Wallis test** | | | | | |
| --- | --- | --- | --- | --- | --- | --- | --- | --- | --- | --- |
|  |  |  |  |  | **Site** | | | **Season** | | |
|  |  |  |  |  | ***df*** | ***X²*** | ***p*** | ***df*** | ***X²*** | ***p*** |
| Electrical conductivity | dS·m⁻¹ | 0.44 ± 0.28  (0.20–0.73) | 0.52 ± 0.16  (0.33–0.71) | 0.46 ± 0.27  (0.21–0.73) | 2 | 0.15 | 0.926 | 3 | 5.67 | 0.129 |
| pH | pH scale | 5.50 ± 0.80  (4.7–6.7) | 4.60 ± 0.10  (4.5–4.8) | 5.6 ± 0.8  (4.8–6.7) | 2 | 6.58 | **0.037*** | 3 | 1.36 | 0.715 |
| Total lime | % CaCO₃ | 3.00 ± 1.00  (2.1–4.5) | 2.3 ± 0.90  (1.7–3.6) | 3.0 ± 0.9  (2.0–4.3) | 2 | 1.88 | 0.390 | 3 | 2.59 | 0.459 |
| Organic matter | % | 24.8 ± 1.33  (23.2–26.2) | 81.20 ± 1.19  (79.9–82.8) | 24.2 ± 1.87  (22.2–26.5) | 2 | 7.42 | **0.024*** | 3 | 3.00 | 0.392 |
| Practical salinity | PSU | 0.19 ± 0.12  (0.09–0.32) | 0.23 ± 0.07  (0.14–0.31) | 0.20 ± 0.12  (0.09–0.32) | 2 | 0.15 | 0.926 | 3 | 5.67 | 0.129 |

**Table S3.** Results of Dunn’s test for pairwise comparisons of pH and organic matter between the three study sites. Significant differences are indicated by an asterisk (*).

|  | **Site 1** | **Site 2** | ***n*1** | ***n*2** | **Statistic** | ***p*** | **Adjusted *p*** |
| --- | --- | --- | --- | --- | --- | --- | --- |
| pH | El Mellah | Righia | 4 | 4 | -2.06 | **0.039*** | 0.118 |
| pH | El Mellah | Verges | 4 | 4 | 0.29 | 0.769 | 1 |
| pH | Righia | Verges | 4 | 4 | 2.35 | 0.018* | 0.055 |
| Organic matter | El Mellah | Righia | 4 | 4 | 2.26 | **0.024*** | 0.072 |
| Organic matter | El Mellah | Verges | 4 | 4 | -0.19 | 0.845 | 1 |
| Organic matter | Righia | Verges | 4 | 4 | -2.45 | **0.014*** | **0.042*** |

**Table S4.** Correlation coefficients and significance values for soil variables and non-metric multidimensional scaling (NMDS) axes. Significant differences are indicated in bold.

|  | **NMDS1** | **NMDS2** | ***R^2^*** | ***p*** |
| --- | --- | --- | --- | --- |
| Electrical conductivity | -0.374 | -0.927 | 0.067 | 0.736 |
| pH | -0.113 | 0.993 | 0.191 | 0.406 |
| Total lime | 0.649 | 0.759 | 0.180 | 0.419 |
| Organic matter | 0.193 | -0.981 | 0.705 | **0.003**** |
| Practical salinity | -0.381 | -0.924 | 0.067 | 0.735 |
